# Enhancing infrared emission of mercury telluride (HgTe) quantum dots by plasmonic structures

**DOI:** 10.1038/s41377-020-0276-1

**Published:** 2020-03-10

**Authors:** Shaofan Yuan, Chen Chen, Qiushi Guo, Fengnian Xia

**Affiliations:** 1 Department of Electrical Engineering, Yale University, New Haven, CT 06511 USA; 2 Department of Electrical Engineering, California Institute of Technology, Pasadena, CA 91125 USA

**Keywords:** Quantum dots, Nanophotonics and plasmonics, Quantum dots, Nanophotonics and plasmonics, Quantum dots

## Abstract

The coupling of HgTe quantum dots to a gold nanobump plasmonic array can enhance the spontaneous infrared emission by a factor of five and reduce the influence of nonradiative decay channels.

The demand for efficient, room-temperature shortwavelength infrared (SWIR) emitters is increasing since they can find important applications in night vision, healthcare, spectroscopy, and light detection and ranging (LIDAR) systems^1^. Despite their tremendous technical importance, there are still challenges associated with the material synthesis and light emission efficiency. First, there are very limited material choices for the fabrication of SWIR light emitters. Although narrow-bandgap III–V compounds can cover this wavelength range^2^, these materials are usually grown epitaxially on lattice-matched substrates, which is an expensive process^2^. Second, the narrow bandgap makes the spontaneous light emission process inefficient—the excited electrons are susceptible to nonradiative transitions such as defect and infrared phonon scattering, thus compromising the spontaneous light emission efficiency^2,3^.

Quantum dots (QDs) provide an alternative avenue for the realization of tunable narrow-bandgap materials. Due to strong quantum confinement, QDs can exhibit a larger bandgap than their bulk counterparts^4^ (see Fig. 1a). By controlling their physical sizes, tunable emission at infrared wavelengths can be realized in mercury telluride (HgTe) QDs, which is a semimetal in its bulk form^5^. In addition, the spontaneous emission rate is not necessarily a fixed material property but can also be controlled by its local electromagnetic environment^6,7^. The modification of the spontaneous emission of emitters by engineering the electromagnetic environment was originally proposed by Purcell in 1946^8^ and was first experimentally demonstrated by Drexhage in 1970^9^. In a recent publication, A. A. Sergeev and coworkers leveraged nanobump plasmonic resonator arrays and significantly enhanced the spontaneous emission of HgTe QDs^10^. Specifically, a HgTe QD and organic dodecanethiol (DDT) ligand layer was coated on a glass-supported gold plasmonic nanobump-array film, which was fabricated through melt and resolidification induced by a femtosecond laser.

**Fig. 1:**
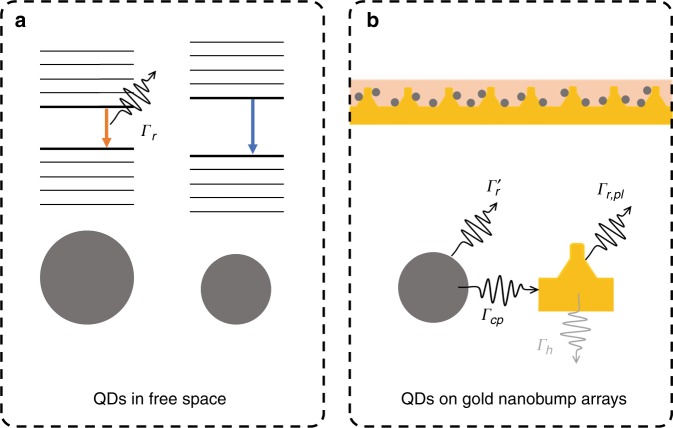
Schematics of the coupling between quantum dots (QDs) and gold nanobump arrays. **a** The band gap increases with a decrease in the size of the QDs. QDs in free space spontaneously emit at a rate of *Γ*_*r*_. **b** Schematics of gold nanobump arrays and the coupling between QDs and the nanobump. After coupling to the external resonate structures, the QDs directly emit into free space at a modified rate of $\varGamma_r^\prime$. More importantly, the QDs can excite resonate modes of the structure at a larger rate of *Γ*_*cp*_ due to the enhanced electromagnetic local density of states (LDOS). The energy of these resonant modes can be either emitted into free space at a rate of *Γ*_*r,pl*_ or dissipated at a rate of *Γ*_*h*_ as heat. This figure is drawn based on the device concept in ref. ^10^

Let us assume that the spontaneous emission rate of an emitter in free space is *Γ*_*r*_ (Fig. 1a) without surrounding photonic structures. When the emitter is coupled to resonant structures, such as nanobump resonators (Fig. 1b), the emitter can excite resonant modes of the structure at a rate of *Γ*_*cp*_. In a judiciously designed resonant structure, *Γ*_*cp*_ can be much larger than the original *Γ*_*r*_ due to the greatly enhanced local density of states^11^. The resonant structure then radiates into free space at a rate of *Γ*_*r,pl*_, or the energy can dissipate as heat at a rate of *Γ*_*h*_ (Fig. 1b). Along with coupling to the resonant structure, the emitter is also directly emitted into free space at a modified rate of $\varGamma_{\it{r}}^\prime$. Considering all of the factors above, $\varGamma_{far} = \varGamma_r^\prime + \varGamma_{cp}\varGamma_{r,pl}/\left(\varGamma_{r,pl} + \varGamma_h \right)$ is the overall far-field emission rate^6^. Therefore, an enhancement in the spontaneous emission (*Γ*_*far*_ > *Γ*_*r*_) can be observed when the energy is efficiently transferred from the emitter to the resonant structure (high *Γ*_*cp*_) and then effectively radiated into free space (high *Γ*_*r,pl*_/(*Γ*_*r,pl*_ + *Γ*_*h*_)).

In the work by A. A. Sergeev and coworkers, two types of pyramid-shaped QDs were synthesized with average sizes of 3.9 and 5.0 nm, exhibiting spontaneous emission peaks at 1.6 and 2.2 µm, respectively^10^. By tuning the periodicity of the nanobumps, the spontaneous emission of QDs matches the first-order resonance wavelength of the plasmonic arrays, giving rise to an emission enhancement factor of five^10^. The observed photoluminescence (PL) decay rate was also faster than the decay rate of QDs on a smooth silicon substrate without cavities, which confirmed the enhanced radiative rate enabled by the plasmonic nanobump arrays^10^. Moreover, an average four-fold enhancement of absorption was also achieved because of the strong local field in these plasmonic nanobump arrays^10^. The integration of HgTe QDs with designed nanophotonic structures offers a new route to on-chip, compact and tunable SWIR light emission devices, which may find various applications in spectroscopy and sensing. As a result, this demonstration represents an important step towards the realization of future on-chip infrared photonic systems.
